# Rewiring the Obsessive-Compulsive Disorder Brain: Mechanisms and Clinical Implications of Deep Brain Stimulation-Induced Neuroplasticity

**DOI:** 10.1192/j.eurpsy.2026.11747

**Published:** 2026-07-31

**Authors:** O. Scheuermann, E. Jetter, D. Valle, C. Angelle, B. Carr

**Affiliations:** 1 Department of Psychiatry; 2 College of Medicine, University of Florida, Gainesville, United States

## Abstract

**Introduction:**

Deep Brain Stimulation (DBS) in psychiatry is increasingly understood as a network-level intervention, extending beyond localized modulation to large-scale neural circuit reorganization. This makes it especially useful for psychiatric disorders, such as obsessive-compulsive disorder (OCD), in which dysfunctional connectivity in networks such as the Default Mode Network (DMN), salience network, and frontostriatal circuits can contribute to difficulty with self-focus, rumination, lack of cognitive flexibility, and compulsive behaviors.

**Objectives:**

To discuss the mechanisms by which DBS for OCD induces functional network reorganization and structural adaptation to facilitate long-term neuroplasticity, rather than acting as a static intervention. To assess the clinical implications that DBS for OCD can have, given its potential as a long-term intervention.

**Methods:**

A literature review was performed, looking at data largely published between 2020-2025, to synthesize current evidence from neuroimaging, electrophysiology, and clinical studies to characterize the impact of DBS on functional connectivity, oscillatory dynamics, and structural remodeling of the brain in patients with refractory OCD. Key networks of interest included the DMN, salience network, and frontostriatal control circuits.

**Results:**

Overall, DBS targets maladaptive neural pathways in OCD by reducing hyperconnectivity within the DMN, enhancing salience network flexibility, and strengthening dorsolateral prefrontal cortex–striatal regulation of compulsive behaviors. Functional MRI studies of DBS treated patients demonstrate reconfiguration of resting-state networks, promoting balance between cognitive control, emotion regulation, and self-referential processing. Mechanistically, DBS exerts long-term effects via activity-dependent remodeling, enhancing white matter integrity in tracts (anterior limb of internal capsule, medial forebrain bundle, and corpus callosum), recalibrating inappropriately hyperconnected regions, and reorganizing oscillatory rhythms across theta, beta, and gamma bands. Clinically, these adaptive changes underpin sustained therapeutic benefits, provide potential biomarkers of DBS response (i.e. gray matter changes, white matter coherence), and enable personalized modulation strategies.

**Conclusions:**

DBS is not merely a symptomatic intervention but a driver of long-term neuroplasticity. By reconfiguring structural and functional connectivity within critical neural networks, DBS supports sustained therapeutic benefits in psychiatric disorders. Recognizing these adaptive changes can guide personalized modulation strategies, enhance biomarker development, and refine patient selection criteria.

**Disclosure of Interest:**

None Declared

